# Supplementary material for: hoxc12/c13 as key regulators for rebooting the developmental program in Xenopus limb regeneration
Source: Nat Commun. 2024 Apr 22;15:3340. doi: 10.1038/s41467-024-47093-y (PMC11035627; doi:10.1038/s41467-024-47093-y)
Supplement: Supplementary file 1 — Supplementary Information [file 41467_2024_47093_MOESM1_ESM.pdf]

# Supplementary Information for

## *hoxc12/c13* as key regulators for rebooting the developmental program in *Xenopus* limb regeneration

Aiko Kawasumi-Kita, Sangwoo Lee, Daisuke Ohtsuka, Kaori Niimi, Yoshifumi Asakura, Keiichi Kitajima, Yuto Sakane, Koji Tamura, Haruki Ochi, Ken-ichi T Suzuki, and Yoshihiro Morishita*

*Corresponding author. Email: yoshihiro.morishita@riken.jp

**This PDF file includes:**

Supplementary Figures 1 to 16
Supplementary Table 1

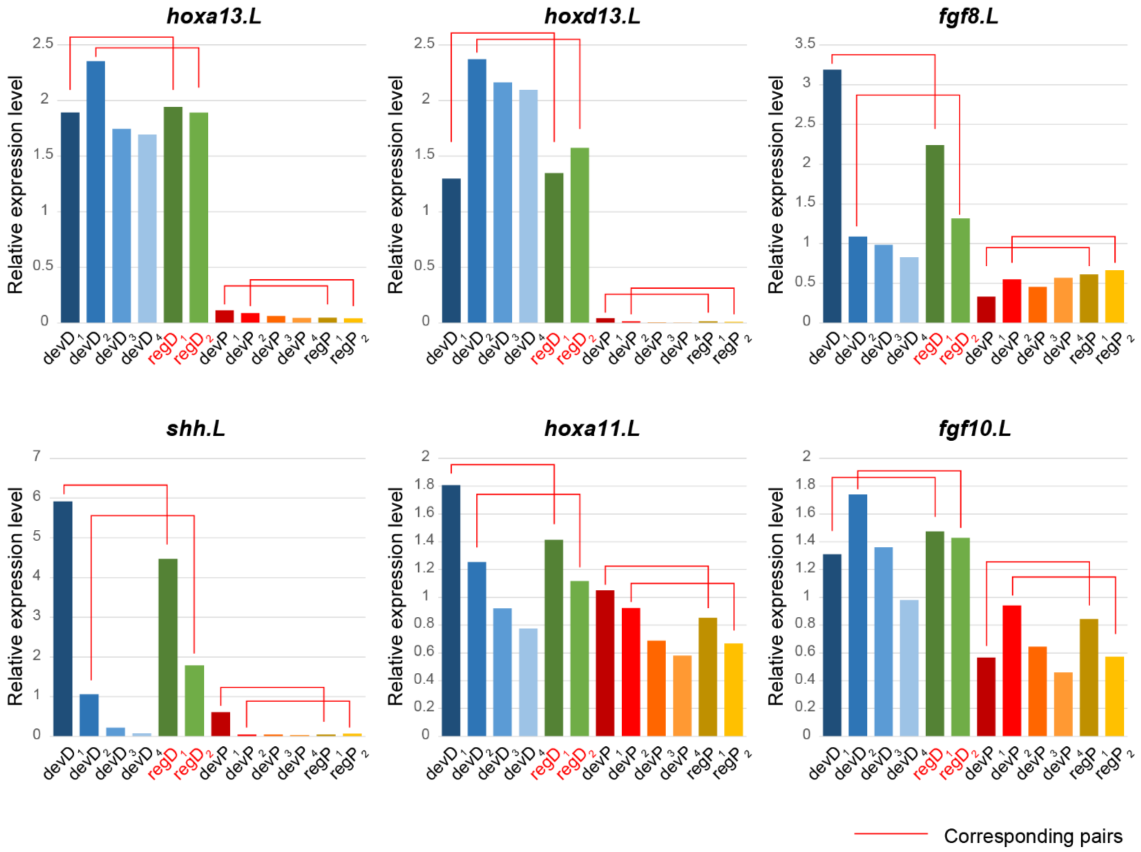


**Fig. S1. Results of transcriptomic analysis for typical patterning genes essential for normal limb development**

Relative expression levels of the patterning genes are comparable for each corresponding pair (the means of triplicate data). Source data are provided as a Source Data file.

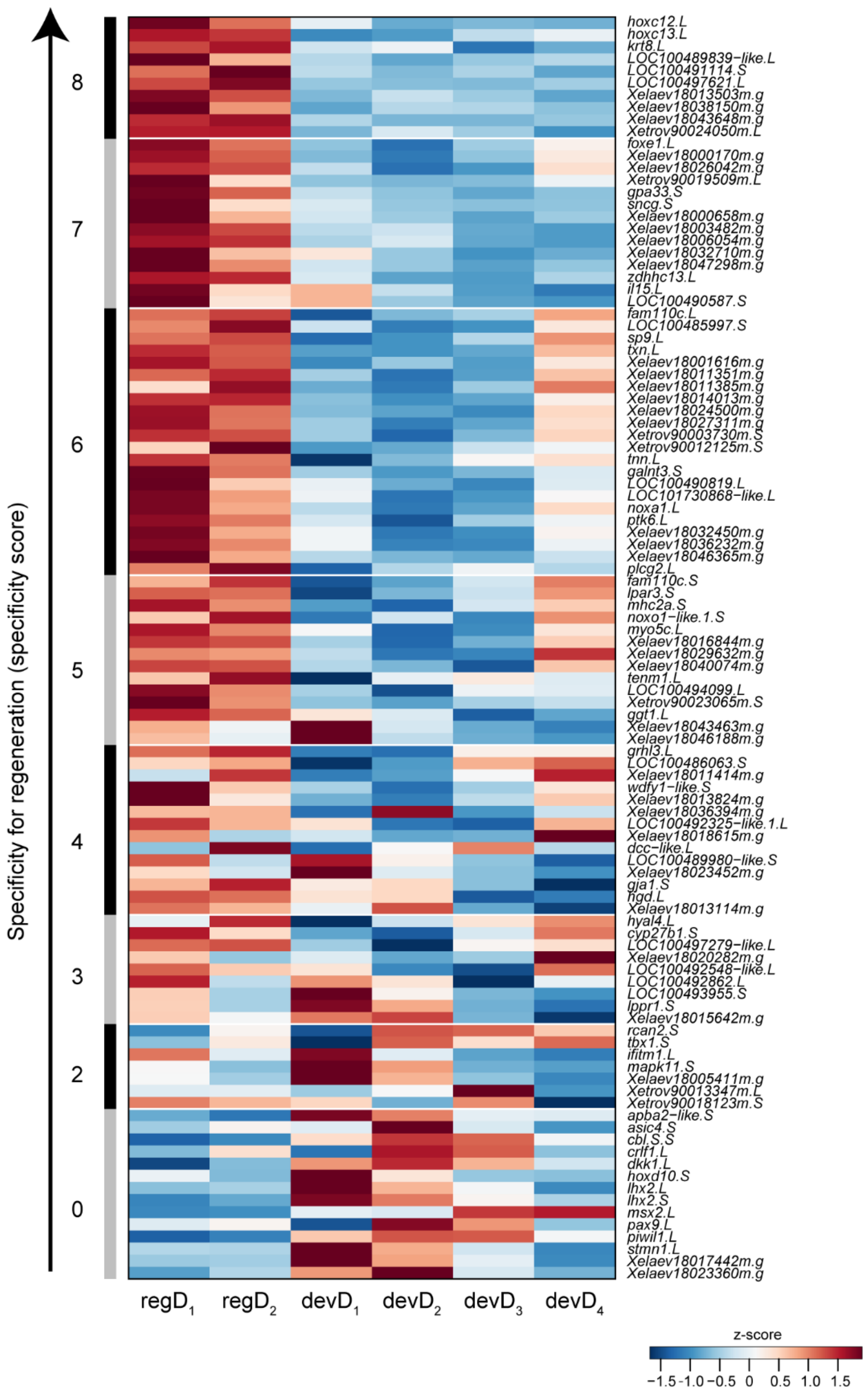


**Fig. S2. List of genes remaining after the second screen in the transcriptomic analysis**

The genes remaining after the second screen are arranged in order of their specificity scores (see also Figs. 1**e-f**).

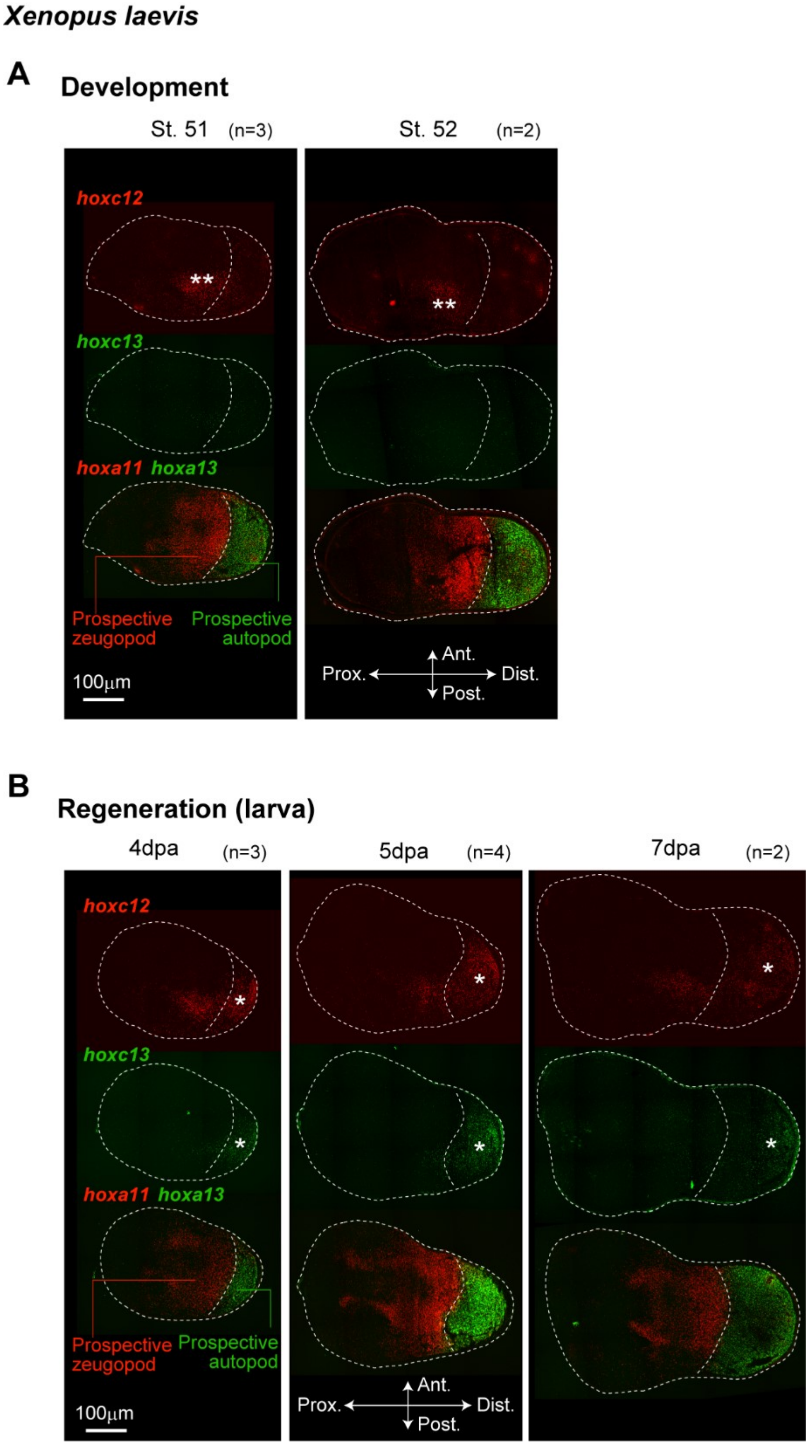


**Fig. S3. *hoxc12/c13* expression patterns during limb development and regeneration**
(**A**) *hoxc12/c13* expression patterns in the developing limb buds at St. 51 and 52, and (**B**) in the larval regenerating limb blastema at 4, 5, and 7 dpa. Each experiment was independently repeated for each gene with similar results.

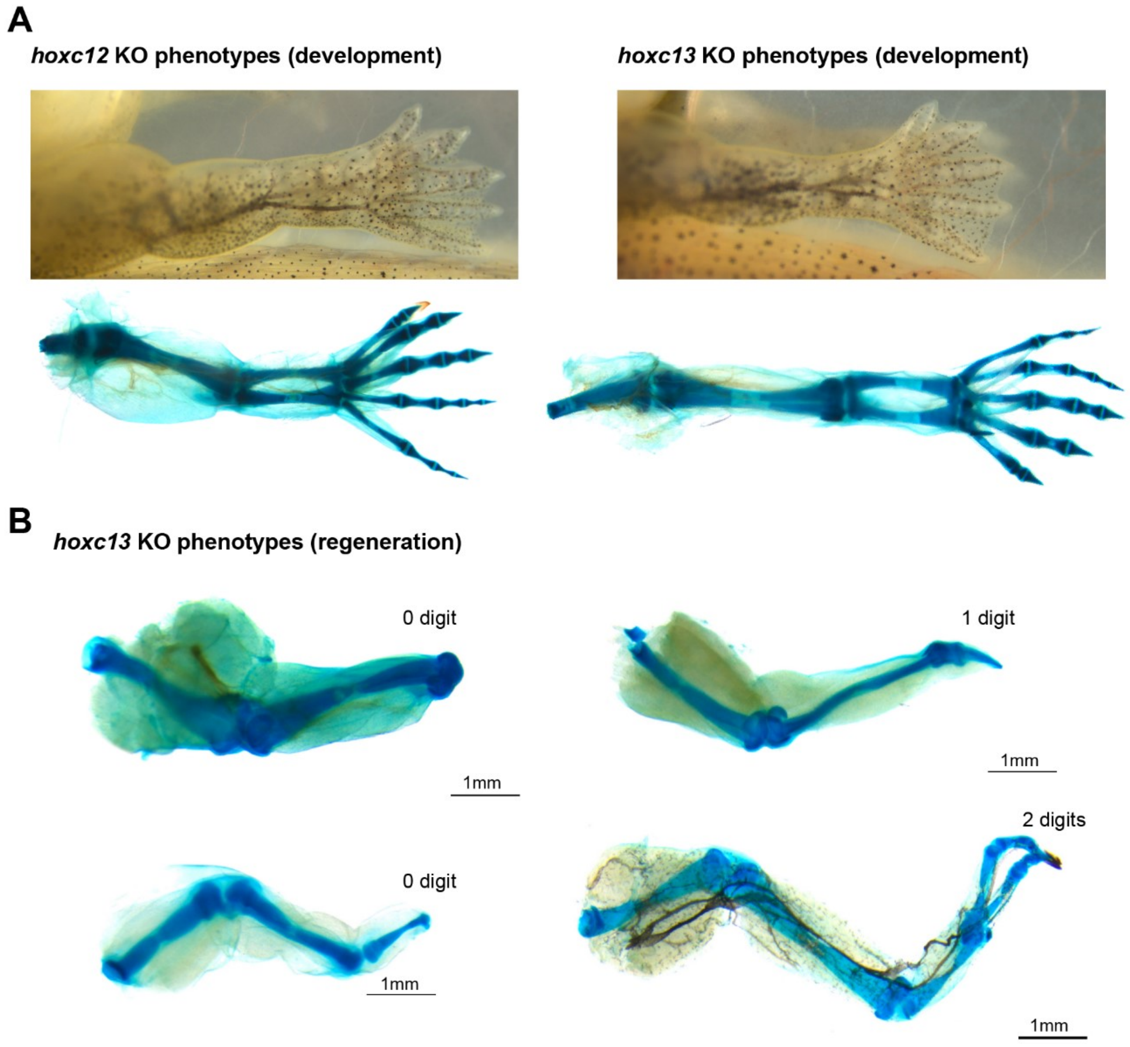


**Fig. S4. Effects of *hoxc12*/*c13* knockout on larval limb development and regeneration**

Examples of limb morphologies/cartilage patterns in development **(A)** and in regeneration **(B).** Cartilage was stained with Alcian blue. *hoxc12*-KO: *hoxc12*$^{-/-}$; *hoxc13*-KO: *hoxc13*$^{-/-}$.

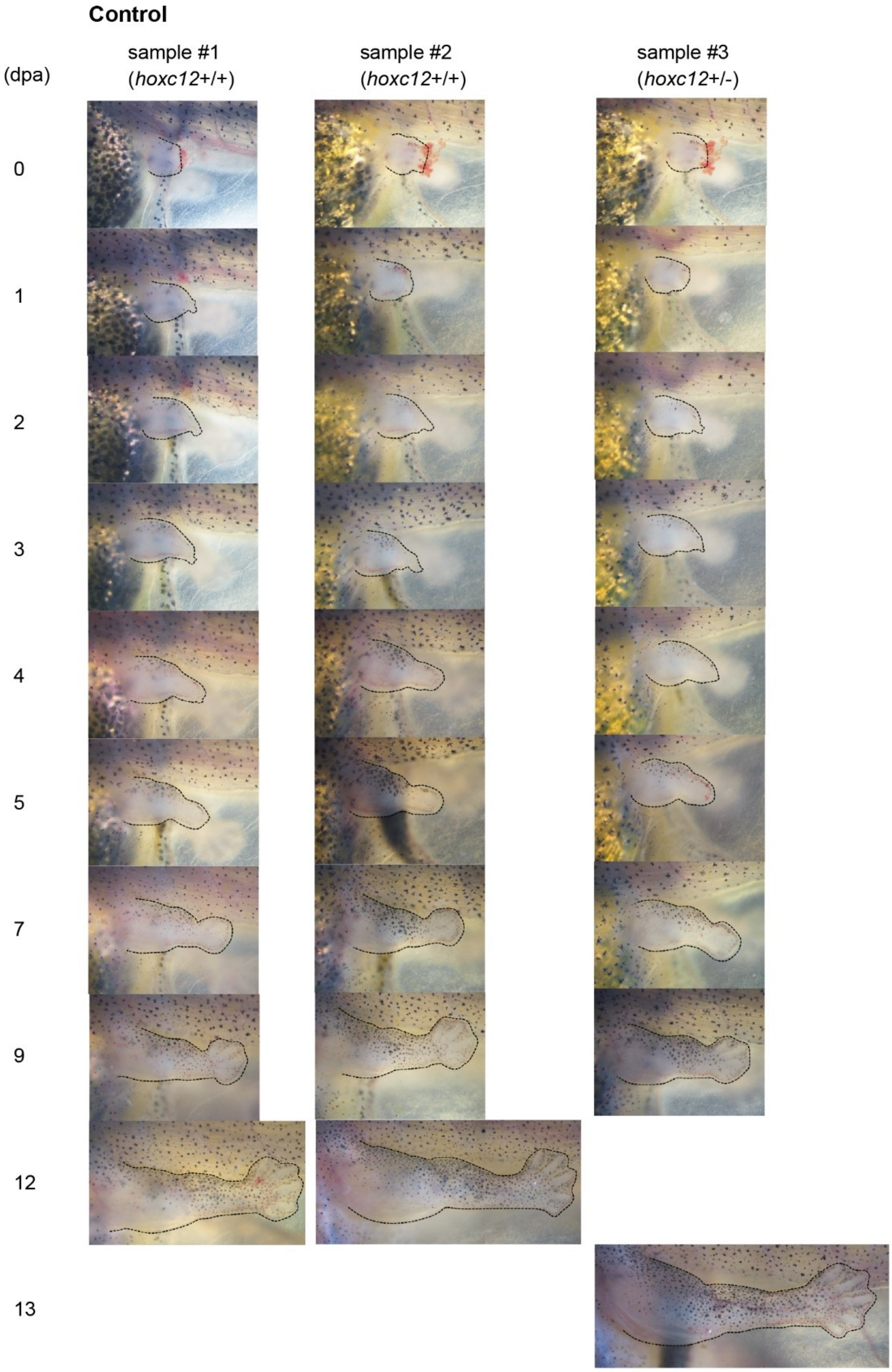


**Fig. S5. Time course of regenerating limb blastema in wild type individuals**

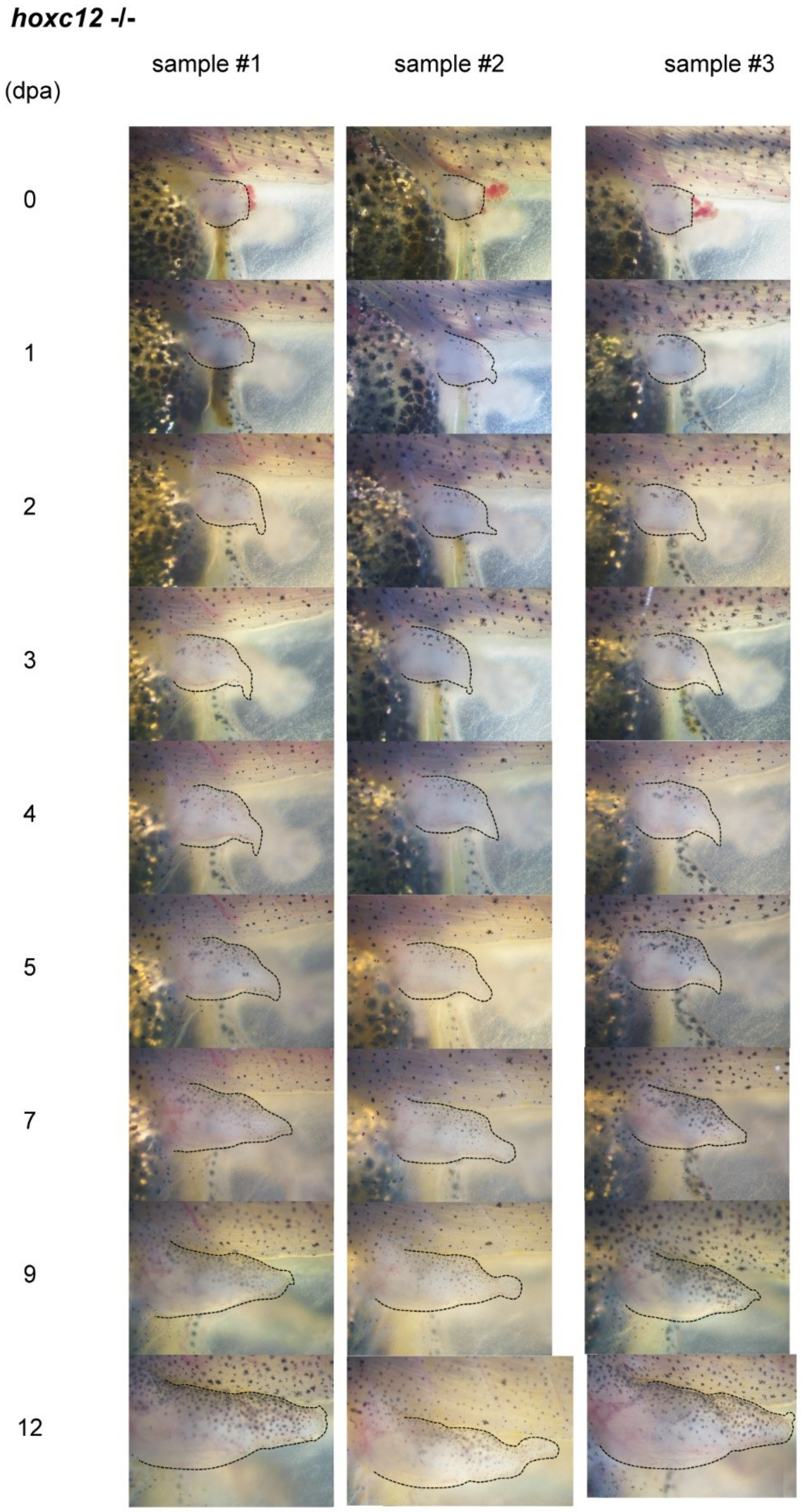


**Fig. S6. Time course of regenerating limb blastema in *hoxc12* $^{-/-}$ individuals**

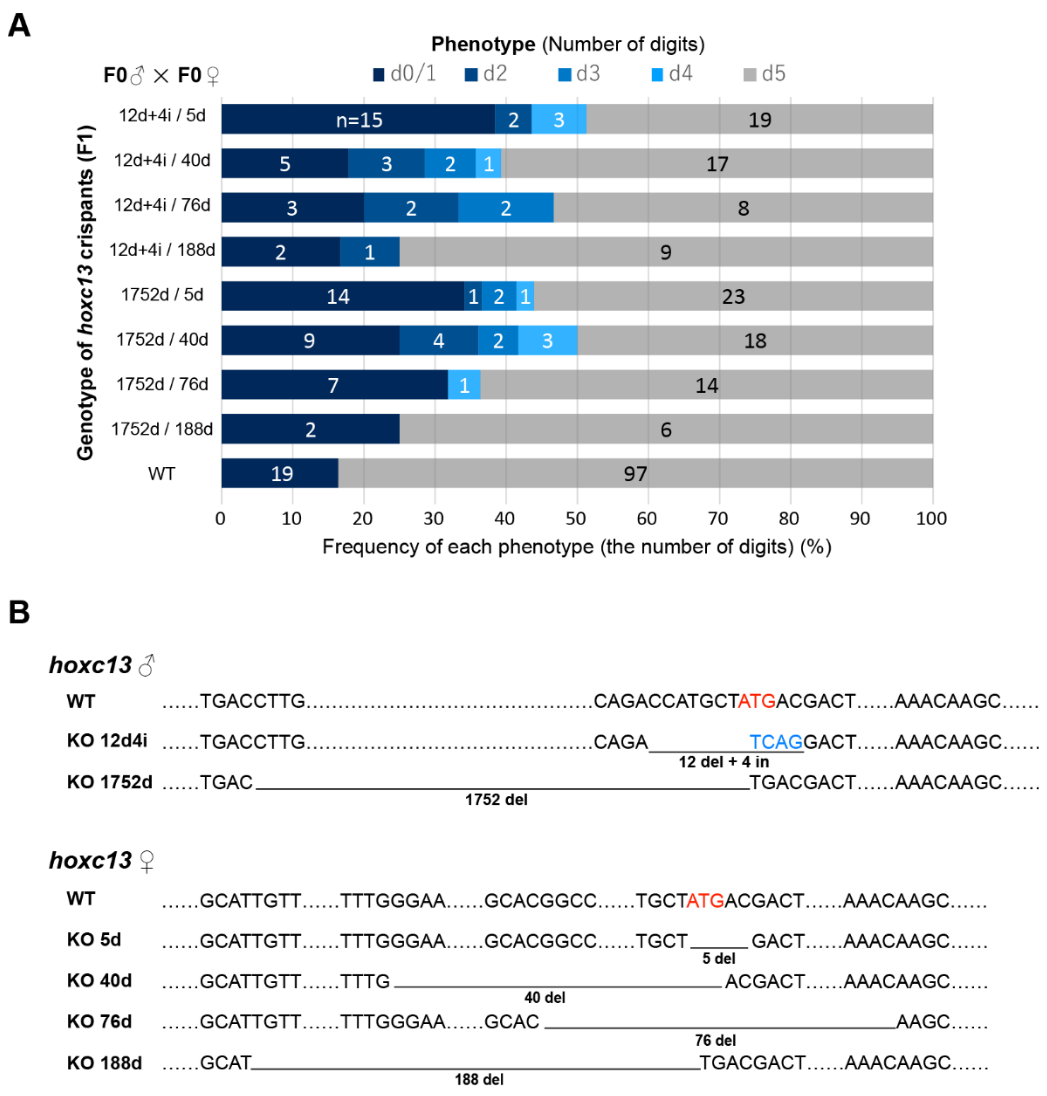


**Fig. S7. Effects of *hoxc13* knockout by genome editing in larval limb regeneration**

(**A**) Frequency distribution of phenotypes defined by the number of digits for individuals with deletions or insertions of different sub-sequences by genome editing. (**B**) Information about the deletion or insertion of sub-sequences for each parent.

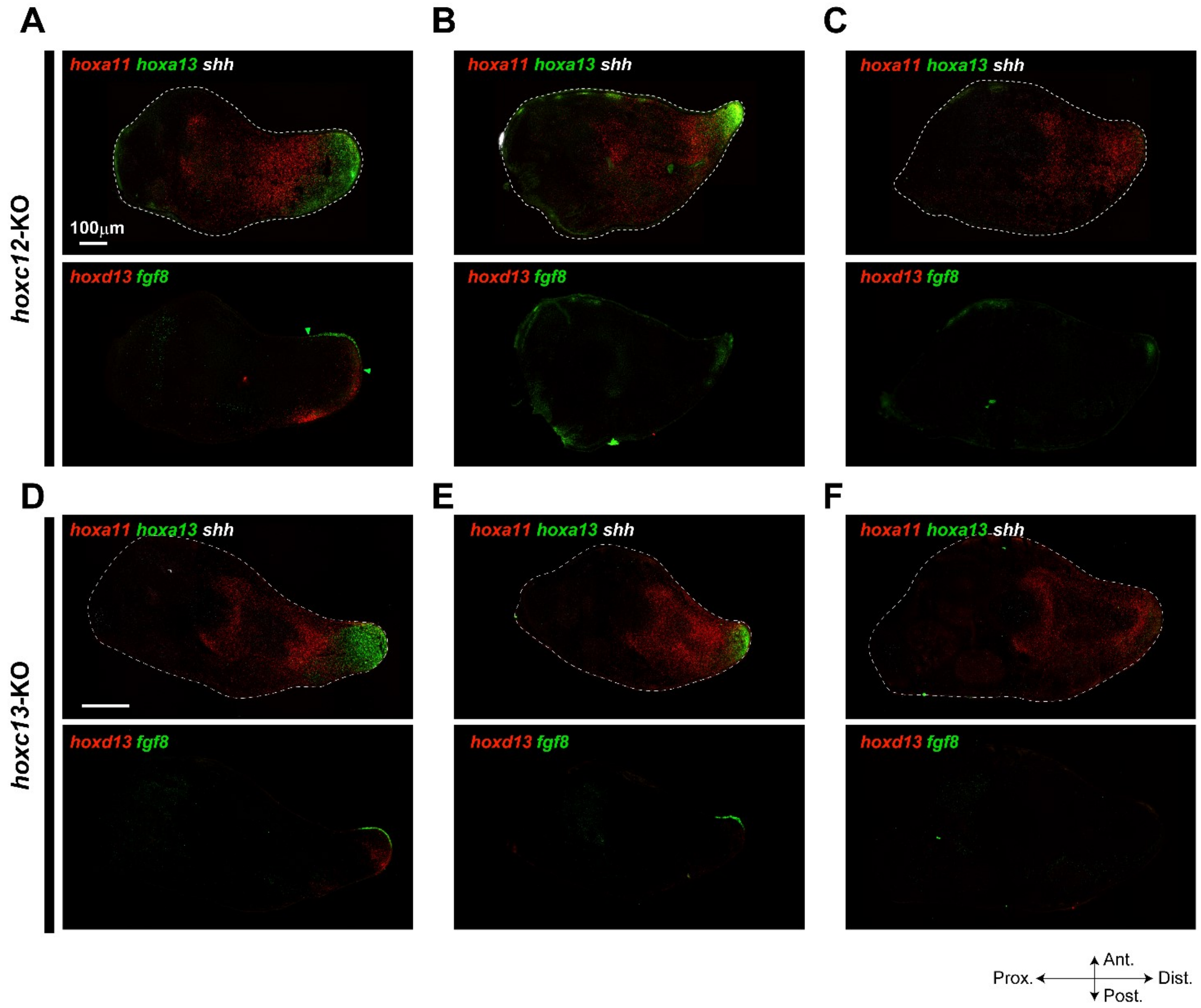


**Fig. S8. RNAscope results for pattering genes (6 examples in addition to those shown in Fig.3a)**

(**A**, **D**) Mild morphological defects. *hoxa11* and *hoxa13* were exclusively expressed along the PD axis. shh expression was not detected. *fgf8* expression was observed in the anterior half of the distal epithelium. The expression range of *hoxd13* was smaller than during normal development (Fig. 3**a**). (**B**, **E**) Blastema growth was severely reduced. *hoxa13* was expressed at the distalmost region, but no exclusive expression with *hoxa11* was observed. *shh*, *hoxd13* expression was almost undetectable. *fgf8* expression was almost undetectable in (**B**) and was observed in the anterior half of the distal epithelium in (**E**). (**C**, **F**) Blastema growth was also severely reduced. Expression of genes other than *hoxa11* was almost undetectable.

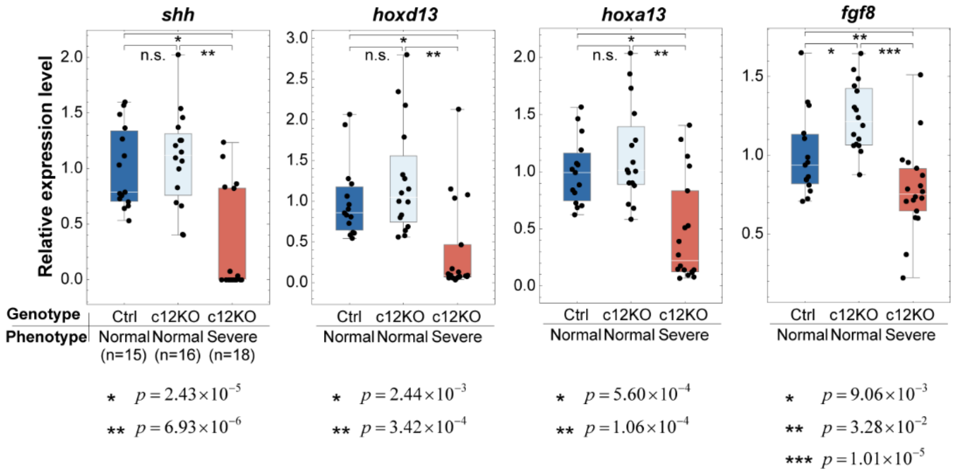


**Fig. S9. Result of qPCR assay for some patterning genes.** Comparisons were made among control (*hoxc12*+/-, with normal phenotype) and *hoxc12*-KO (with normal or severe phenotype). Box-plot elements: center line, median; box limits, upper and lower quartiles; whiskers, max/min. Statistical test (Student's t-test, two-sided). Source data are provided as a Source Data file.

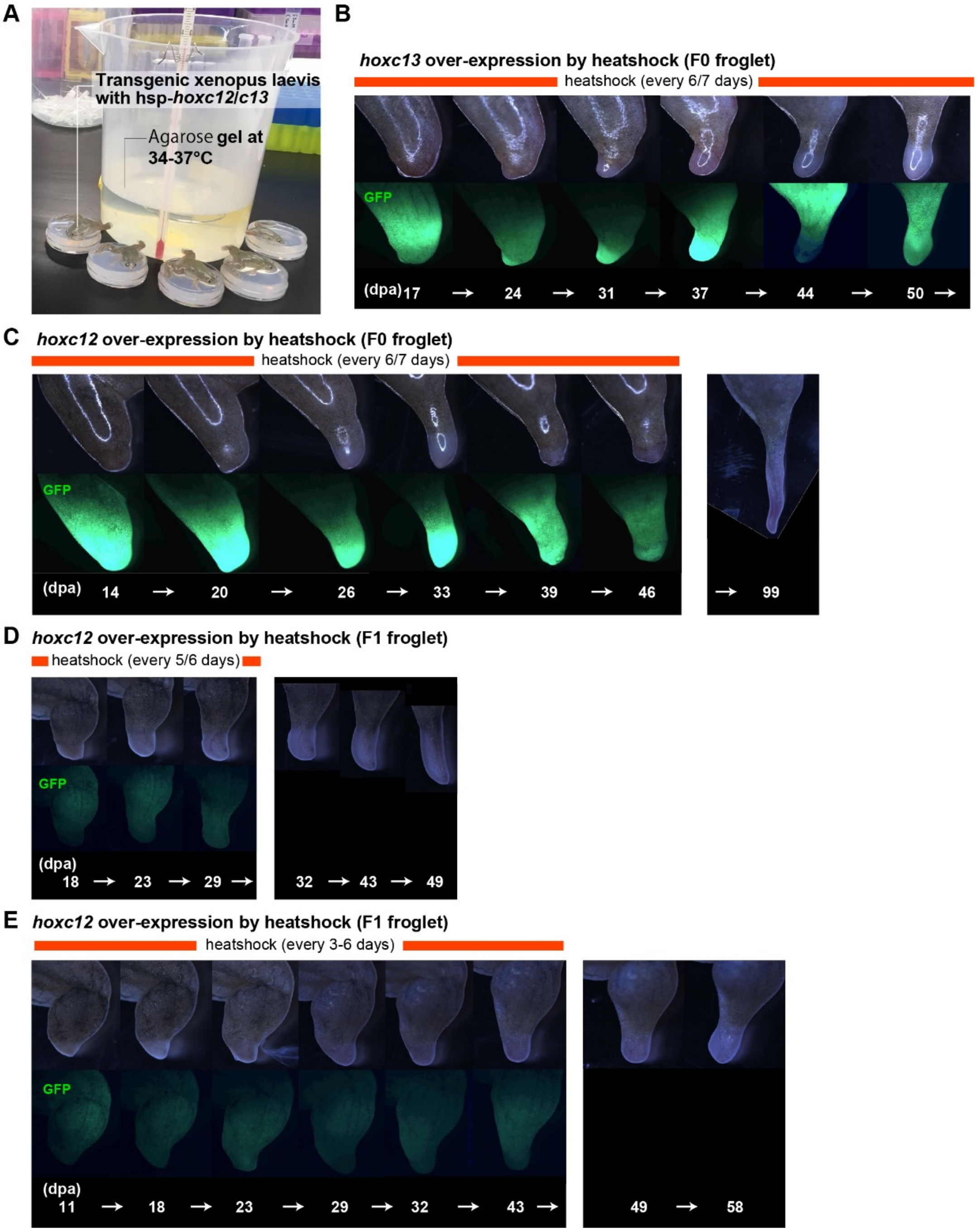


**Fig. S10. Heat-shock induction of *hoxc12* or *c13* expression and phenotypes**

(**A**) Photo showing the experimental set up for the heat-shock induction of *hoxc12* or *hoxc13* expression by embedding amputated hindlimbs in agarose gel at 34-37°C (see Methods). (**B**-**E**) Morphological changes in blastema with *hoxc12* or *hoxc13* overexpression induced by heat-shock at varying durations (4 examples in addition to that shown in Fig. 4**b**). It should be noted that the induced GFP level by heat-shock in F1 individuals are weaker than that in F0, but still stronger than that by amputation stress only, for which microscopic detection was difficult.

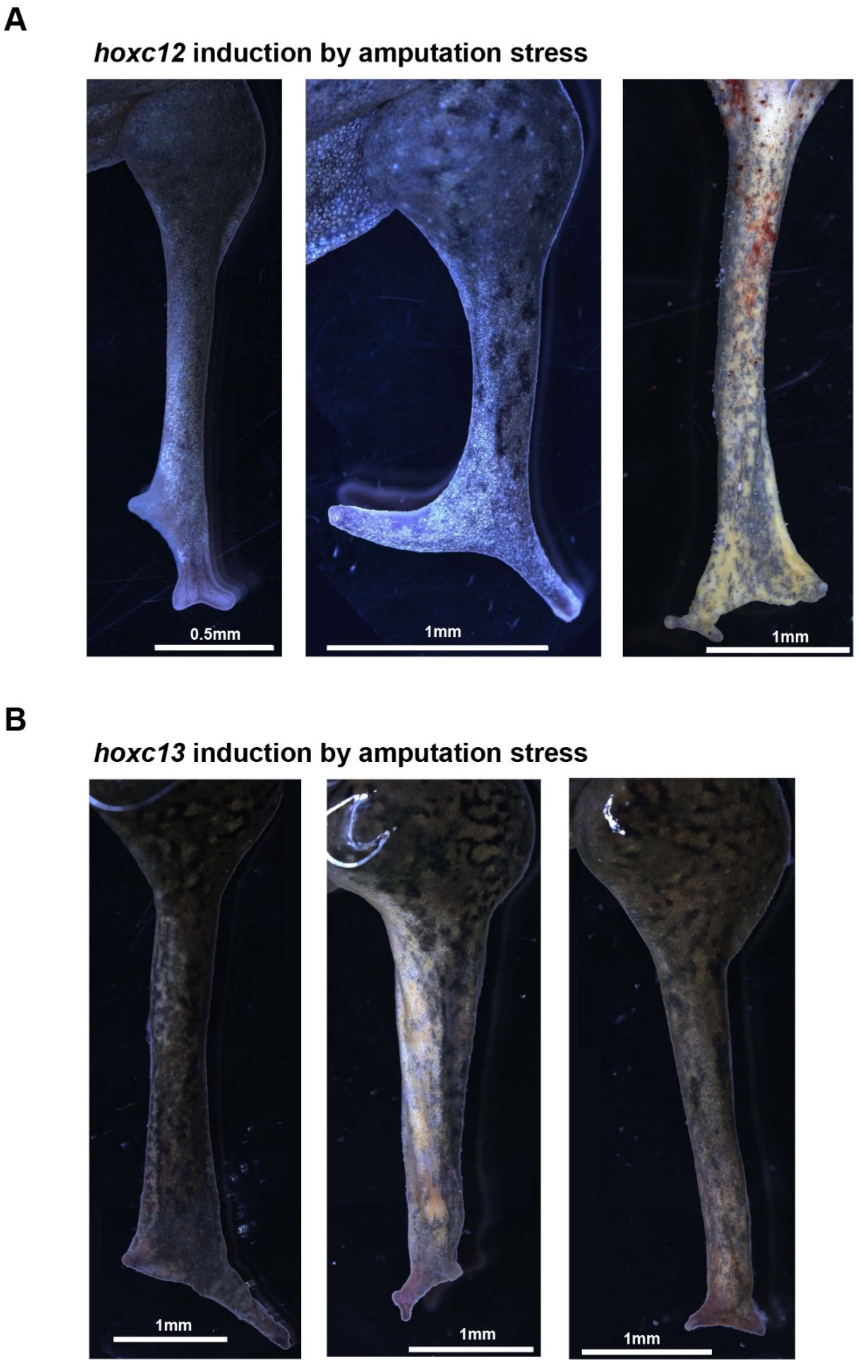


**Fig. S11. Regenerated limbs with multifurcated distal cartilage**

Phenotypes generated by (**A**) *hoxc12* or (**B**) *hoxc13* induction by amputation stress (without heat-shock).

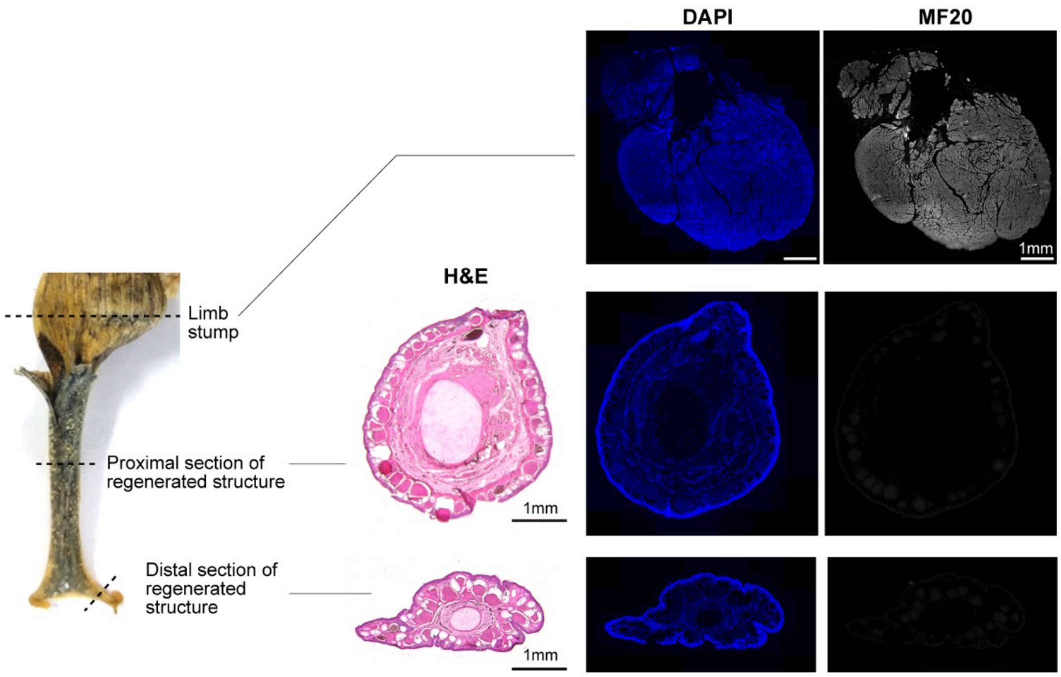


**Fig. S12. Muscle staining using MF20.** No muscle was observed within the regenerated limb structures of Tg individuals.

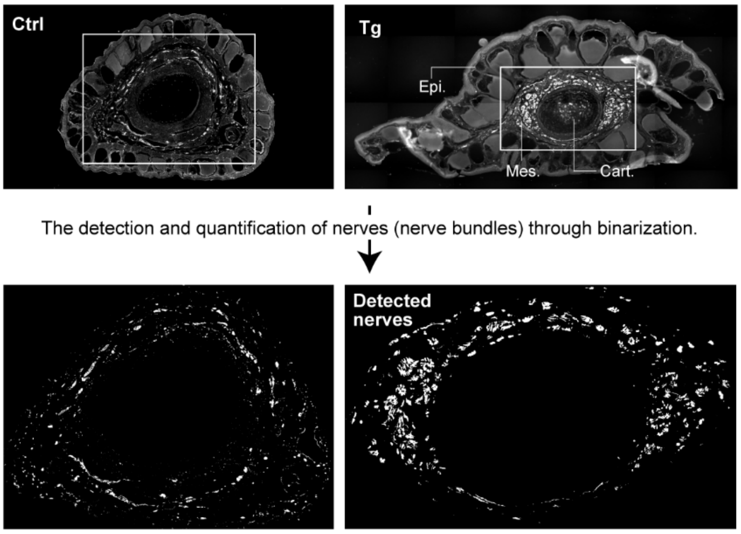


**Fig. S13. Image analysis for detecting nerves (acetylated tubulin signal).**

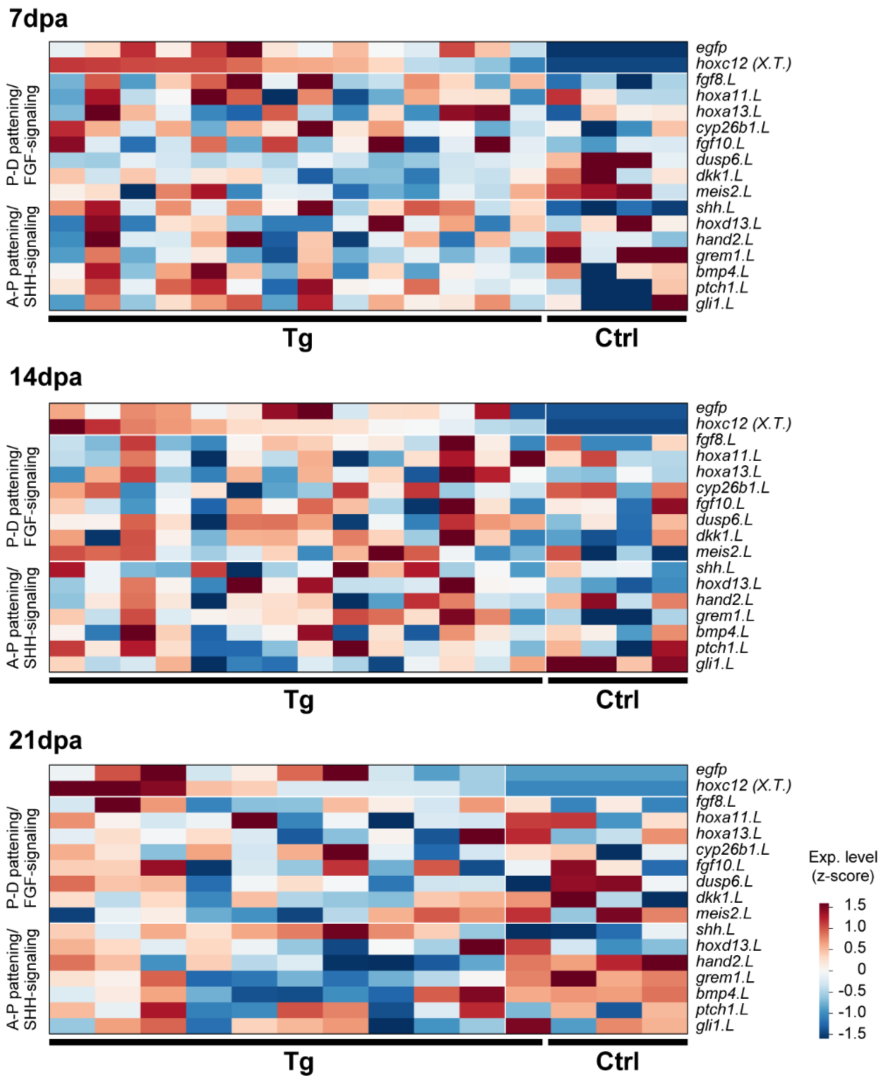


**Fig. S14. Comparative analysis of transcriptome data (froglet regeneration at 7, 14, 21 dpa) for typical patterning genes that function during limb development**

Source data are provided as a Source Data file.

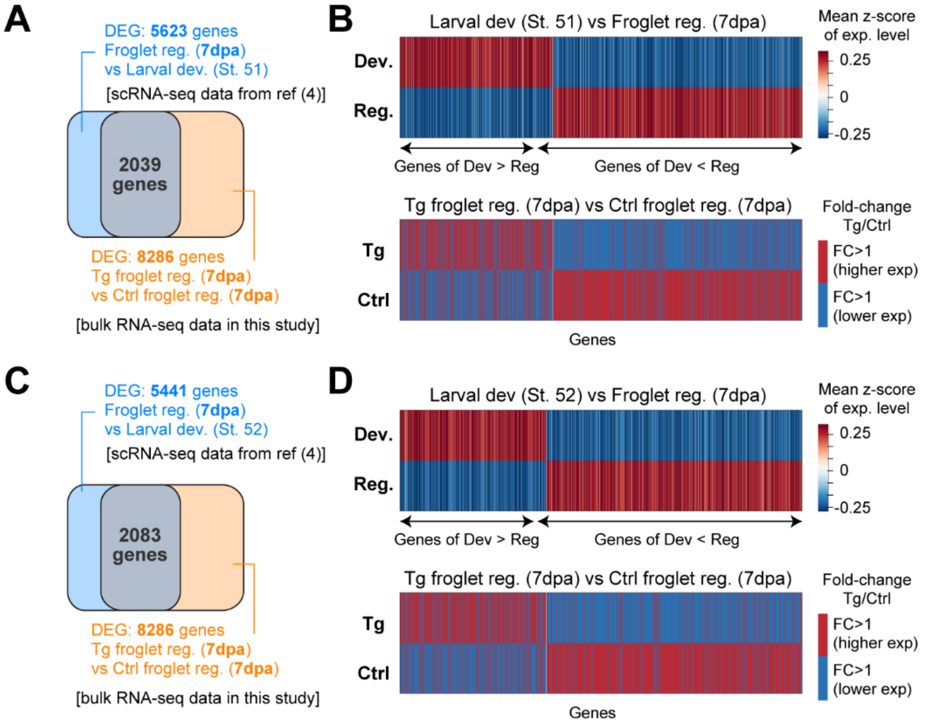


**Fig. S15. Comparative transcriptome analysis.** (**A**, **C**) Extraction of differentially expressed genes (DEGs) between developing limb buds (**A**: St. 51; **C**: St.52) and froglet blastema (7dpa) based on single cell transcriptome data from Lin et al (2021) ($DEG_{bl\text{-}dev}$), and DEGs between *hoxc12*Tg/control froglet blastema (7dpa) based on bulk-transcriptome data obtained in this study ($DEG_{Tg}$). (**B**, **D**) Comparison of expression levels for genes that are common in both $DEG_{bl\text{-}dev}$ and $DEG_{Tg}$, which are identified in **A** and **C**, respectively; (top) developing limb bud vs froglet blastema and (bottom) Tg vs control froglet blastema. Source data are provided as a Source Data file.

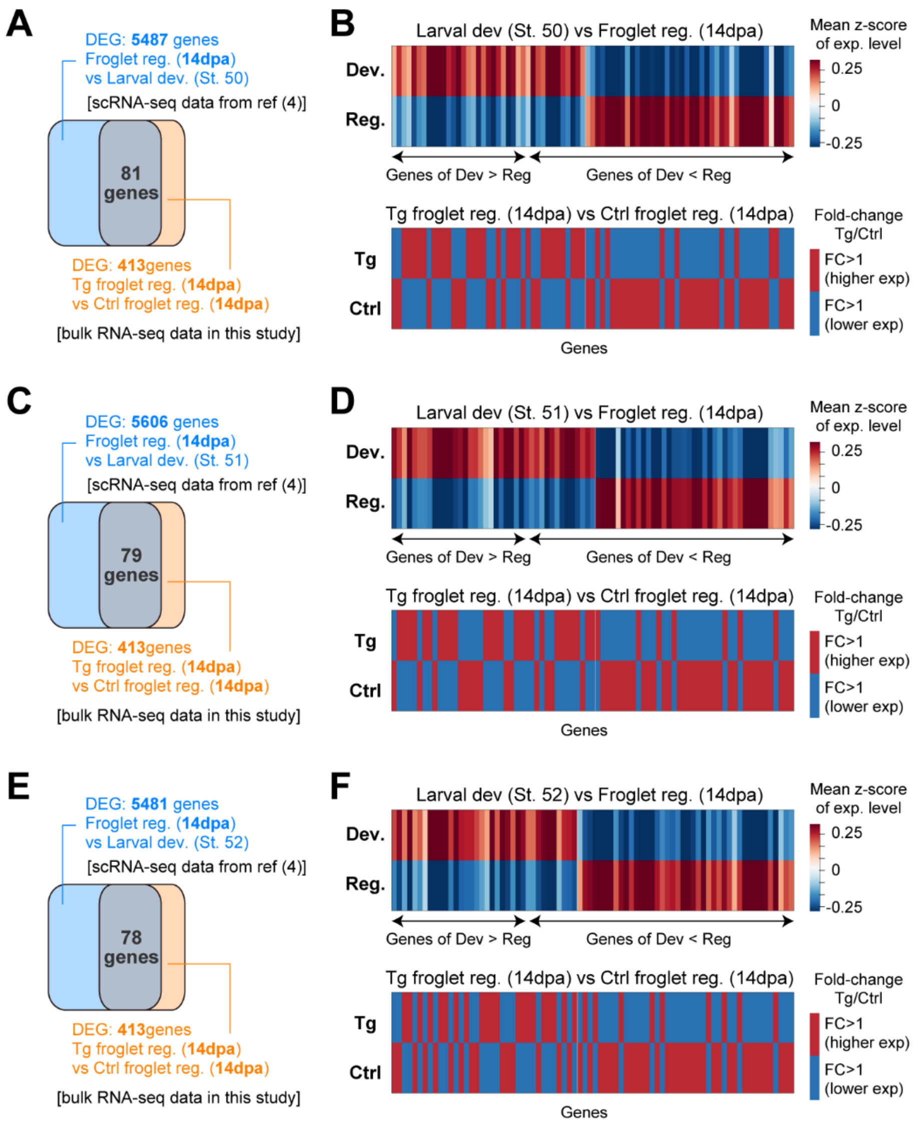


**Fig. S16. Comparative transcriptome analysis.** (**A**, **C, E**) Extraction of differentially expressed genes (DEGs) between developing limb buds (**A**: St. 50; **C**: St. 51, **E**: St. 52) and froglet blastema (14dpa) based on single cell transcriptome data from Lin et al (2021) (DEG$_{\text{bl-dev}}$), and DEGs between *hoxc12*Tg/control froglet blastema (14dpa) based on bulk-transcriptome data obtained in this study (DEG$_{\text{Tg}}$). (**B**, **D, F**) Comparison of expression levels for genes that are common in both DEG$_{\text{bl-dev}}$ and DEG$_{\text{Tg}}$, which are identified in **A**, **C**, and **E**, respectively; (top) developing limb bud vs froglet blastema and (bottom) Tg vs control froglet blastema. Source data are provided as a Source Data file.

| Gene | Forward Primer Sequence | Reverse Primer Sequence | Reporter 1 Sequence |
|---|---|---|---|
| *hoxa11* (*X.trop*) | CGGCAACAATGAGGACAAAGC | CTGGTATTTGGTATAAGGGCACCTT | CTGCCCACTGCTGCTG |
| *hoxa13* (*X.trop*) | CAACCCACCTCTGGAAATCGT | CGAGCTGCTGTCTGACTGATG | CCTGCCGGATGTCGTG |
| *hoxc12.L* (*X.laev*) | CCGACTAATCAAGGCAACAACAGT | TCGGGACCTTGTGTGAATTGG | AAGGAGCCCCACTGGCA |
| *hoxc13.L* (*X.laev*) | CGGTCACTTATGGAAAGCACCTT | CCCCTGCGGTAGTTGCT | CCCAGATGTGGTCCCC |
| *hoxd13* (*X.trop*) | CAAGCCCCACACTTCTGGAA | ACATGTCCGCCTGGTTTAGC | CCTTCCCAGGAGATGTAG |
| *fgf8* (*X.trop*) | GATTAACGCCATGGCAGAAGAC | AACTCTGCTTCCAAACGTATCTGTT | ACCCACATGCCAAGTTA |
| *Shh.L* (*X.laev*) | CGAGTCCAAAGCTCATATTCACTGT | AGCACCCGCCAGACTTG | ACTGAGTTCTCTGCTTTGAC |

**Supplementary Table 1**: The primers and fluorogenic probes for all genes in real-time PCR analysis.
