# Supplementary material for: hoxc12/c13 as key regulators for rebooting the developmental program in Xenopus limb regeneration
Source: Nat Commun. 2024 Apr 22;15:3340. doi: 10.1038/s41467-024-47093-y (PMC11035627; doi:10.1038/s41467-024-47093-y)
Supplement: Supplementary file 3 — Description of Additional Supplementary Files [file 41467_2024_47093_MOESM3_ESM.pdf]

Description of Additional Supplementary Files

Supplementary Data1:

Gene Ontology annotations for the 104 genes (shown in Fig. 1f) selected through transcriptome analysis.

Supplementary Movie1:

An example of regenerated structure after hindlimb amputation in *hoxc12* transgenic Xenopus laevis, with trifurcation of cartilage at the distal tip.
