# Supplementary material for: hoxc12/c13 as key regulators for rebooting the developmental program in Xenopus limb regeneration
Source: Nat Commun. 2024 Apr 22;15:3340. doi: 10.1038/s41467-024-47093-y (PMC11035627; doi:10.1038/s41467-024-47093-y)
Supplement: Supplementary file 6 — Reporting Summary [file 41467_2024_47093_MOESM6_ESM.pdf]

Corresponding author(s): Yoshihiro Morishita

Last updated by author(s): Feb 22, 2024

Reporting Summary

Nature Portfolio wishes to improve the reproducibility of the work that we publish. This form provides structure for consistency and transparency in reporting. For further information on Nature Portfolio policies, see our Editorial Policies and the Editorial Policy Checklist.

Statistics

For all statistical analyses, confirm that the following items are present in the figure legend, table legend, main text, or Methods section.

n/a	Confirmed	
☐	☒	The exact sample size (*n*) for each experimental group/condition, given as a discrete number and unit of measurement
☐	☒	A statement on whether measurements were taken from distinct samples or whether the same sample was measured repeatedly
☐	☒	The statistical test(s) used AND whether they are one- or two-sided<br>*Only common tests should be described solely by name; describe more complex techniques in the Methods section.*
☒	☐	A description of all covariates tested
☒	☐	A description of any assumptions or corrections, such as tests of normality and adjustment for multiple comparisons
☐	☒	A full description of the statistical parameters including central tendency (e.g. means) or other basic estimates (e.g. regression coefficient) AND variation (e.g. standard deviation) or associated estimates of uncertainty (e.g. confidence intervals)
☐	☒	For null hypothesis testing, the test statistic (e.g. *F*, *t*, *r*) with confidence intervals, effect sizes, degrees of freedom and *P* value noted<br>*Give P values as exact values whenever suitable.*
☒	☐	For Bayesian analysis, information on the choice of priors and Markov chain Monte Carlo settings
☒	☐	For hierarchical and complex designs, identification of the appropriate level for tests and full reporting of outcomes
☒	☐	Estimates of effect sizes (e.g. Cohen's *d*, Pearson's *r*), indicating how they were calculated

Our web collection on statistics for biologists contains articles on many of the points above.

Software and code

Policy information about availability of computer code

Data collection	No software was used for data collection.
Data analysis	Python (v3.12.2)<br>R (v4.2)<br>FASTX Toolkit (v0.0.14)<br>Bowtie (v2.2.2)<br>TopHat (v2.0.11)<br>Cuffdiff (v2.2.1)<br>edgeR (v3.8.6)<br>scikit-learn library (v1.3)<br>org.Xl.eg.db package (v3.16.0)<br>GO.db package (v3.16.0)<br>Trim_galore (v0.6.10)<br>STAR (v2.7.9a)<br>featureCounts v2.0.1<br>Seurat v4.4.0<br>DESeq2 v3.18

For manuscripts utilizing custom algorithms or software that are central to the research but not yet described in published literature, software must be made available to editors and reviewers. We strongly encourage code deposition in a community repository (e.g. GitHub). See the Nature Portfolio guidelines for submitting code & software for further information.

Data

Policy information about availability of data

All manuscripts must include a data availability statement. This statement should provide the following information, where applicable:

- Accession codes, unique identifiers, or web links for publicly available datasets
- A description of any restrictions on data availability
- For clinical datasets or third party data, please ensure that the statement adheres to our policy

The raw sequence reads generated in this study have been deposited in the NCBI Sequence Read Archive (SRA) under the accession number SRP349043 [https://www.ncbi.nlm.nih.gov/sra/?term=SRP349043].
Source data are provided with this paper.
We also used single-cell transcriptome data GEO: GSE165901.

Research involving human participants, their data, or biological material

Policy information about studies with human participants or human data. See also policy information about sex, gender (identity/presentation), and sexual orientation and race, ethnicity and racism.

Reporting on sex and gender	N/A
Reporting on race, ethnicity, or other socially relevant groupings	N/A
Population characteristics	N/A
Recruitment	N/A
Ethics oversight	N/A

Note that full information on the approval of the study protocol must also be provided in the manuscript.

Field-specific reporting

Please select the one below that is the best fit for your research. If you are not sure, read the appropriate sections before making your selection.

☒ Life sciences ☐ Behavioural & social sciences ☐ Ecological, evolutionary & environmental sciences

For a reference copy of the document with all sections, see nature.com/documents/nr-reporting-summary-flat.pdf

Life sciences study design

All studies must disclose on these points even when the disclosure is negative.

Sample size

In the bulk-transcriptome analysis shown in Fig. 1 and Fig. 3d, we used replicate numbers of 3 or 4 to ensure reproducibility of results. In the bulk-transcriptome analysis, we used a higher number of replicates (n=15, for the data of transgenic animals) because not all individuals exhibited the phenotype with cartilage branching and we were unsure if the transgene (hoxc12) would be expressed due to limb amputation stress alone in all individuals. We did not employ strict statistical methods to determine the sample size, but given the high reproducibility of the results, we considered these sample sizes to be sufficient. For other experiments, we selected the sample size to ensure data distribution for each group. No data were excluded from the analyses, and animals were selected at random for imaging and gene expression analysis. We did not specifically distinguish between the sexes of the animals. This is because there is no evidence of gender-based differences in regenerative capacity.

Data exclusions

No data were excluded from the analyses.

Replication

Bulk transcriptome
(1)Fig.1, Xenopus laevis, development vs larval regeneration (3 replicates, independent samples)
(2)Fig.3C-D,Xenopus tropicalis, larval regeneration, hoxc13KO vs control (4 replicates, independent samples)
(3)Fig.5, Xenopus laevis, froglet regeneration, hoxc12Tg vs control (15 replicates for hoxc12Tg and 4 replicates for control, independent samples)

Fig1H, RNAscopes, development(n=3); regeneration(n=3), biologically independent samples
Fig2B, phenotyping, WT(n=157); hoxc12-KO(n=98); hoxc13-KO(n=69), biologically independent samples
Fig2D, msx1 expression, control(n=4); hoxc12-KO(n=5); hoxc13-KO(n=3), biologically independent samples
Fig3A-B, RNAscope, control(n=19); hoxc12-KO(n=19); hoxc13-KO(n=22), biologically independent samples
Fig3E, pH3, control(n=9); hoxc12-KO_normal(n=12); hoxc13-KO_normal(n=10); hoxc12-KO_severe(n=7); hoxc13-KO_severe(n=7), biologically independent samples

Fig4D, hoxc12Tg(n=30); hoxc13Tg(n=21)
Fig4E, pH3, control(n=10); hoxc12-Tg(n=10), biologically independent samples
Fig4H, n=4 for each, biologically independent samples

Randomization	Embryos were selected at random for imaging and gene expression analysis.
Blinding	N/A

Reporting for specific materials, systems and methods

We require information from authors about some types of materials, experimental systems and methods used in many studies. Here, indicate whether each material, system or method listed is relevant to your study. If you are not sure if a list item applies to your research, read the appropriate section before selecting a response.

Materials & experimental systems

n/a	Involved in the study
☐	☒ Antibodies
☒	☐ Eukaryotic cell lines
☒	☐ Palaeontology and archaeology
☐	☒ Animals and other organisms
☒	☐ Clinical data
☒	☐ Dual use research of concern
☒	☐ Plants

Methods

n/a	Involved in the study
☒	☐ ChIP-seq
☒	☐ Flow cytometry
☒	☐ MRI-based neuroimaging

Antibodies

Antibodies used	Primary antibodies<br>anti-phosphorylated Histone H3 (PH3) antibody (Millipore, 06-570)<br>anti-acetylated tubulin antibody (Sigma-Aldrich, T7451)<br>anti-myosin heavy chain (DSHB, MF20)<br><br>Secondary antibody<br>Goat anti mouse IgG (H+L) cross absorbed secondary antibody, AlexaFluor 633 (Invitrogen, A-21050)
Validation	We have only used antibodies that have been previously validated for efficacy.

Animals and other research organisms

Policy information about studies involving animals; ARRIVE guidelines recommended for reporting animal research, and Sex and Gender in Research

Laboratory animals	(1) Xenopus laevis (wild type): purchased from a domestic animal vendor;<br>(2) Xenopus laevis (transgenic): we generated them by modifying the animals provided from Dr. Hitoshi Yokoyama in Hirosaki University, Japan;<br>(3)Xenopus tropicalis: provided by National BioResource Project (in Hiroshima University, Japan)<br><br>Ages:<br>- Xenopus laevis larval limb development: tadpoles at St. 51, 52, 52.5, 53, 54 were used.<br>- Xenopus laevis larval limb regeneration: tadpole limbs were amputated at St. 52.<br>- Xenopus laevis froglet limb regeneration: several months individuals were used.<br>- Xenopus tropicals larval limb regeneration: tadpole limbs were amputed at St. 52.
Wild animals	N/A
Reporting on sex	At the present time, as there is no evidence of gender-based differences in limb regeneration ability, animals were randomly selected regardless of sex.
Field-collected samples	The study did not involve samples collected from the field.
Ethics oversight	Experiments on Xenopus limb development were conducted at Hiroshima University, Yamagata University, and RIKEN Center for Biosystems Dynamics Research (RIKEN BDR), Japan. Procedures and protocols were approved by the Institutional Animal Care and Use Committee of Hiroshima University. RIKEN and Yamagata University (as well as Japanese domestic law, according to the Act on Welfare and Management of Animals) exempt studies involving amphibians from requiring IRB approval, however, all experiments at RIKEN BDR and Yamagata University were performed in accordance with the principle of 3R (Replacement, Reduction, and

Refinement).

Note that full information on the approval of the study protocol must also be provided in the manuscript.
